# Investigation of the effect of flipped listening instruction on the listening performance and listening anxiety of Chinese EFL students

**DOI:** 10.3389/fpsyg.2022.1043004

**Published:** 2022-12-02

**Authors:** Yu Qiu, Wei Luo

**Affiliations:** ^1^School of Foreign Studies, Lingnan Normal University, Zhanjiang, China; ^2^School of Foreign Languages, Anyang University, Anyang, China

**Keywords:** flipped instruction, listening performance, listening anxiety, IELTS, EFL

## Abstract

**Introduction:**

Given the fact that flipped instruction especially with the aid of technology has gained momentum in second language (L2) instruction, numerous L2 researchers have explored the usefulness of flipped classroom for L2 learning.

**Methods:**

As an attempt to further this research area, the current research examined the effect of flipped listening instruction on the Chinese English as a foreign language (EFL) students’ listening performance and listening anxiety using a quasi-experimental research design. To this end, a total number of 44 EFL learners from two intact classes in a Chinese language school were selected as the participants of the research and they were randomly assigned as the control group (*N* = 21) and an experimental group (*N* = 23). Within a course of one semester, the control group was instructed employing traditional listening instruction, while the experimental group were taught based on the flipped mode of instruction.

**Results:**

The data collection was carried out by administering the listening section of IELTS and Foreign Language Listening Anxiety Scale (FLLAS).

**Discussion:**

The results of ANCOVA revealed that the flipped listening instruction significantly enhanced listening performance of the participants. Also, the flipped classroom substantially reduced listening anxiety of the EFL learners. The outcomes of this research might provide notable implications for EFL practitioners.

## Introduction

The upsurge of technology has led to dramatic change in education (Nickerson and Zodhiates, 2013). With the advent of the internet during the 1990s, accompanied by Web 2.0, higher education has dramatically shifted from traditional pedagogical methods to current models using computer technology (Valtonen et al., 2021). E-learning as a new technological innovation is increasingly used in educational contexts. The widespread availability of authentic resources is one of the biggest advantages of the technology for language learners (Yunus, 2018). In the meantime, the recent developments in technological devices (e.g., mobile technology and smartphones) have led to ever-increasing popularity of computer-assisted language learning (CALL) as a mode of learning and instruction in educational contexts (Chen et al., 2021; Lim and Aryadoust, 2021; Park and Son, 2022; Rahimi and Fathi, 2022). As a result of such developments, blended learning has gained more recognition nowadays (Liu, 2020). Blended learning is regarded as a fundamental redesign of the instructional method with a paradigm shift from lecture-centered to learner-centered instruction where learners have active roles in their learning process (Poon, 2013).

The flipped classroom is a good example of a blended learning methodology which might provide the productive use of face-to-face instruction time with learners (Strelan et al., 2020; Liu et al., 2022). The essence of the flipped instructional approach has been directed toward the development of learning outside the class *via* the visualization of digital content produced by the instructor (Bergmann and Sams, 2012). Consequently, instructors are able to scaffold and guide students’ learning process while providing valuable feedback inside the class.

Learners tend to be more active in flipped classroom because they become independent with the help of their teacher as a facilitator and the guidance of technology tools (Zainuddin and Attaran, 2016). In order to learn effectively in FL classrooms, learners should have the opportunity to engage in a variety of activities to gain a grasp of the new target language. Nonetheless, instructors might inevitably skip important aspects of effective FL teaching because of the restricted classroom time, thus limited time is left for practices (Turan and Akdag-Cimen, 2020). The flipped classroom model also known as inverted classroom switches the classroom structure from a conventional lecture approach to more collaborative learning activities where teachers are able to provide students with additional practices in the classroom (Milman, 2012).

Against this backdrop, the flipped teaching approach has been increasingly integrated into English language classrooms (Fathi and Rahimi, 2020; Afzali and Izadpanah, 2021; Xue and Dunham, 2021; Khazaie et al., 2022; Liu et al., 2022; Teo et al., 2022). It is argued that the flipped instruction is successful in achieving the instructional goals and enhances the EFL learners’ motivation, making them more active in learning activities (Chen Hsieh et al., 2017). From EFL students’ perspectives, using technology in the flipped classroom is an enjoyable and practical way to preview and review materials (Chen Hsieh et al., 2017). Moreover, EFL students appreciate the self-paced and autonomous learning feature of flipped approach since it leads them dive into the content deeply (Aghaei et al., 2020). Overall, students have shown positive attitudes toward language learning in the flipped classroom (Doman and Webb, 2017).

EFL literature includes numerous studies on the use of flipped classrooms in Asian countries (e.g., Turan and Goktas, 2016; Chen Hsieh et al., 2017); however, these studies are predominantly concerned with exploring the perceptions toward flipped classroom, various approaches in using this instruction, and conducting descriptive studies (Doman and Webb, 2014). Furthermore, few studies have explored the effectiveness of flipped instruction using experimental research designs (Webb and Doman, 2016). Moreover, most of the previously conducted studies have focused on investigating the effect of flipped classroom on students’ English skills such as speaking (Abdullah et al., 2021), reading (Samiei and Ebadi, 2021), and writing (Fathi and Rahimi, 2020), few studies thus far have explored the role of flipped approach in listening skill of EFL students. As such, the exploration of how flipping a course can contribute to enhancing EFL students’ listening comprehension might be an interesting research area, particularly given the fact that students’ further control over their learning content is argued to affect their degree of engagement and learning outcomes (Ushioda, 2011). In addition, the effect of flipped classroom on listening anxiety has remained under-explored. The role of anxiety in L2 learning, in general, is of paramount importance (Horwitz et al., 1986) and some scholars have considered this construct to be task-or skill-specific (Mac Intyre and Gardner, 1994; Fathi et al., 2020). As such, investigating the effect of flipped classroom on listening anxiety seems much warranted as one key novelty of this research. The only recent study in this respect is the work of Rajabi et al. (2021) which focused on the impacts of the flipped classroom method on classroom anxiety and listening performance of Iranian EFL students. As the flipped classroom approach has turned out to be implemented by a considerable number of researchers recently in the educational systems (Polat and Karabatak, 2021), the need to investigate the effects of flipped approach on language skills in EFL context is significant. Bearing this in mind, further research is required to reveal how the integration of flipped approach can affect students’ listening skill and listening anxiety among Chinese EFL learners. Therefore, the following research questions guided this research:

**Research Question 1:** Does flipped listening instruction significantly enhance EFL learners’ listening performance?

**Research Question 2:** Does flipped listening instruction significantly reduce EFL learners’ listening anxiety?

## Literature review

### Flipped classroom

This research is theoretically grounded in Wen (2008) Output-driven model based on which learners’ requirement for output prompts them to focus on input, and the input then allows the learners to generate more fine-grained output. From this perspective, appropriate engaging input is required to enhance the degree of learners’ intake, output also aid learners in improving students’ competencies, and finally favorable feedback facilitate learning process (Wen, 2015). This model lends itself to the flipped mode of instruction as the learners are first given videos/clips prior to attending the class *via* getting exposed to the input and then they are required to produce quality output during the class time.

The recent deveopments in technology have positively constructed a diversity of language pedagogical approaches, namely CALL, e-learning, and mobile-assisted language learning (MALL), as well as learning management systems (LMS). According to Hill and Hannafin (2001), the dramatic effect of computer technology has altered both the nature of communication and information and resources available in learning/teaching environments. Riding on the tide of rapid developments in digital technologies, many L2 instructors and learners have gone through digital language learning and its applications (Li and Lan, 2021). In many cases, application of mobile technology creates opportunities for collaborative learning, thus this collaboration in language learning opens up chances for learners to practice L2 skills and build new knowledge both inside and outside the classroom (Kukulska-Hulme and Viberg, 2018). Alongside the rise of online learning, one of the approaches that takes advantage of educational technology is flipped classroom approach. The flipped model reverses the places of activities inside (namely, the student note taking and the teacher-led lecture) and outside the classroom such as homework and assignments (Bergmann and Sams, 2012). Since its arrival, attempts have frequently been made to uncover the extent to which flipped approach is effective in affecting student achievement (Zheng et al., 2020; Polat and Karabatak, 2021).

The flipped approach is a relatively new pedagogical model by which the teacher shares predetermined digital materials with learners *via* a platform outside of the classroom, and also teaches a particular topic *via* this additional platform asynchronously by using instructional videos (Bergmann and Sams, 2012). In the first (pre-class learning) phase of the flipped model, learners acquire knowledge by viewing learning materials in a variety of media formats, including podcasts, online or lecture videos, and text-format input materials sent by the instructor prior to class (Bergmann and Sams, 2012). In the second (in-class learning) phase of the flipped approach, the class time is devoted to student-centered active learning tasks, such as role plays, problem solving, interactive lectures, problem solving, laboratory experiments, and group discussions (Chen et al., 2014).

Over the past years, a surge of interest in conducting the flipped classroom method in both K-12 and higher education can be observed (Bergmann and Sams, 2012; Zhu, 2021). A bulk of research has been done to determine the effectiveness of flipped teaching model in this area (see Strelan et al., 2020). One example of such investigation is the comprehensive meta-analysis of the effect of flipped model on students’ performance by (Strelan et al., 2020). They suggested that flipping the classroom provides opportunity for problem solving and active learning. Likewise, Shi et al. (2020) revealed that the flipped classroom approach promotes college students’ cognitive learning. They also found that flipped classroom is more effective when teachers integrate individualized active and interactive pedagogical approaches. Chuang et al. (2018) asserted that the way learners perceive and prepare information before the class time can affect the potential effectiveness of a flipped classroom. In their study, highly motivated students did the quizzes well and therefore benefited most from the flipped learning.

The flipped learning model has received attention and popularity in the field of English Language Teaching (ELT) among researchers and practitioners recently, and there has been a hightened interest in this topic in recent years (Turan and Akdag-Cimen, 2020). Similarly, the flipped classroom has become very popular among researchers in the context of EFL (Shahnama et al., 2021). Flipped classroom can be beneficial for both the teachers and students in FL classes as instructors are able to address all subjects in the given curriculum and focus more on interactive and collaborative activities (Basal, 2015). It also creates autonomous and flexible learning for students since it allows them to learn at their own pace and experience active learning (Amiryousefi, 2017). Research has shown that flipped classroom provides a sequence of instruction that places learners at the heart of their prior experiences and emphasizes the role of collaborative learning that leads students to improve their higher-order thinking skills (Fathi and Rahimi, 2020). The results of Afzali and Izadpanah (2021) study indicated that flipped classroom raised students’ interactivity and participation in addition to their learning motivation. This, in turn, raised their enjoyment in using the learning English grammar model. In a similar vein, the majority of participants of the flipped group in the study conducted by Haghighi et al. (2019) enjoyed learning English in a flipped learning classroom. They showed that these participants were more engaged in the course input and significantly outperformed their peers in the traditional classroom group. The recent study of Öztürk and Çakıroğlu (2021) demonstrated that self-regulated learning strategies in the flipped classroom model positively influenced the development of FL skills. In this line of inquiry, Namaziandost and Çakmak (2020) investigated the relation of self-efficacy and gender with flipped classroom. The findings of their study revealed that flipped model increased self-efficacy among students in the flipped classroom. Females in the flipped classroom exhibited more significant improvements in their self-efficacy than the male participants. This suggests that female students can increase their individual confidence in the flipped classroom while learning English as a FL.

Delving deeply into this area, researchers have examined the effect of flipped classroom on the language skills such as speaking (Phoeun and Sengsri, 2021), reading (Samiei and Ebadi, 2021), and writing (Challob, 2021) in the field of ELT. Among the four skills, listening has gained little attention in the realm of EFL (Namaziandost et al., 2020). There is still insufficient empirical evidence regarding the interplay between listening comprehension and listening anxiety as FL anxiety is a less thoroughly researched skill in general (Elkhafaifi, 2005). According to Amiryousefi (2017), listening and speaking are two difficult skills to master given their interactive nature, thus through flipped classroom teachers can help EFL learners to develop these language skills. The results of Amiryousefi (2017) study showed that EFL learners’ listening and speaking developed substantially in the flipped learning since they were more engaged with materials and tasks outside the class. A similar pattern can be observed in Namaziandost et al. (2020) findings which stressed the effectiveness of flipped instructional model in EFL learners’ listening comprehension.

### L2 listening and anxiety

Listening is believed to be a crucial component of language learning (Vandergrift and Baker, 2018). The significance of listening for L2/FL learning has been highlighted by scholars in the literatute (Feyten, 1991). Listening comprehension is conceptualized as the ability to comprehend the spoken language of native speakers (Mendelsohn, 1994). Hamouda (2013) further stated that listening comprehension refers to the listeners’ ability to understand the speakers’ speech and their ability to repeat the text. As all well-formed English structures are based on a fundamental group of linguistic rules, speakers may also vary acoustically (realizing individual speech sounds) in addition to their semantic and syntactic preferences (Brothers et al., 2019). As a result, this may lead learners to have difficulty in decoding English sentences while listening to native speakers. In contrast with reading comprehension in which language learners are able to manage the input, listeners have less control over correction and repetition since the producer determines the delivery level (Kim, 2000). In the classroom, students need to have good listening comprehension to get engaged actively in the learning process because the information is mostly presented *via* the instructor’s explanation and discussion (Vandergrift, 2007). Moreover, language learners intend to master listening skill in order to understand the target language (L2) speakers and have access to the rich diversity of aural and visual L2 texts through network-oriented multimedia, such as YouTube, blogs, and podcasts (Vandergrift, 2007). Given its complexity, listening is regarded as a difficult skill for students to understand, particularly in learning a FL. In this respect, Arnold (2000) pinpointed that listening induces anxiety in students because it places pressure on them in the input process. Likewise, Chen (2019) underlined the difficult nature of listening comprehension and considered it as a source of anxiety among EFL learners.

Horwitz et al. (1986) defined FL anxiety “a distinct complex of self-perceptions, beliefs, feelings, and behaviors related to classroom language learning arising from the uniqueness of the language learning process” (p: 128). They also highlighted the significant role of anxiety in determining learners’ success or failure when learning a FL. According to Sparks et al. (2000), FL anxiety is the consequence of low performance. FL anxiety seems to be linked with a particular type of language skill, namely listening (Mac Intyre and Gardner, 1994; Zhang, 2013). Elkhafaifi (2005) presented an empirical investigation of the impact of listening anxiety on learners’ listening comprehension. 233 postsecondary learners of Arabic as a FL were the participants of this study. The findings revealed that FL learning anxiety and FL listening anxiety were negatively associated and both had a negative effect on L2 achievement. This suggested that the participants susceptible to high levels of FL learning anxiety experienced higher levels of listening anxiety. It was also found that FL listening anxiety is related but distinct from FL learning anxiety. Similarly, Kim (2000) indicated that listening anxiety is significantly correlated with both general FL anxiety and listening proficiency. Additionally, lack of confidence in listening was found to be the best predictor of listening proficiency.

As pointed out above, the scrutiny of the existing literature demonstrated that flipped instruction could enhance different constructs in EFL setting (e.g., Lee and Wallace, 2018), including L2 listening outcomes. However, reviewing the existing literature shows that the effect of the flipped classroom on listening comprehension is not yet fully examined, legitimizing conducting further empirical studies in this regard. Furthermore, what is not so far clear is the impact of flipped model on listening anxiety among EFL students. Therefore, taking the significance of listening comprehension and anxiety into account, and taking into consideration the research gap which exists in EFL context, it is deemed necessary to explore the effect of flipped classroom on the listening comprehension and listening anxiety of the participants.

## Materials and methods

### Participants

A total number of 44 EFL students of two intact classes from a private language school in China were selected as the participants of this research. As these students were more accessible to the researchers, convenience sampling method was used for selecting the participants who were all female students with their age ranging from 21 to 28 (*M* = 23.24, *SD* = 5.12). The students’ proficiency level was B1 (intermediate) and they had enrolled in an IELTS preparation course whose purpose was to build up the four skills of the participants to get ready to take IELTS. The purpose of this course was particularly to enhance the listening skill of the participants in a period of 3 months. The two intact classes were randomly assigned to a flipped group (*N* = 23) and a non-flipped group (*N* = 21). Both classes were taught by the same instructor who had the experience of teaching EFL based on flipped mode of instruction. However, no student had previously experienced being taught based on flipped instruction. The homogeneity of the two groups concerning the global language proficiency was examined *via* giving Oxford Placement Test (OPT; Allan, 2004). The results of an independent samples t-test demonstrated that there was not any statistically significant difference between the two groups. The students were informed that their participation in the study was voluntary and the informed consents were obtained too. Additionally, the course was an extra-curricular instruction designed for the purpose of this research and was offered as a free course.

### Materials and instruments

Although the scales/tests had been previously used and validated by researchers, a pilot study was carried out to check the face and content validity as well as the reliability of these scales in the context of this study. To this end, the content and items of the tests were checked by two domain experts to ensure the appropriacy of the items. The scales were also given to 12 intermediate Chinese EFL students to measure their internal consistency. The results of this initial piloting showed the acceptable reliability of the scales. The face and content validities were also approved by the domain experts.

#### Listening for IELTS

The coursebook used for both groups was *Listening for IELTS* developed by Aish and Tomlinson (2017). This coursebook intends to prepare IELTS applicants for the listening module of the exam and is used for those who wish to gain the band score of 6–7. It is one of the widely used coursebooks in IELTS preparation classes as it is well-structured, provides a variety of tasks, and includes a complete answer key. This book contains 12 units which cover the potential topics that test takers might encounter on the IELTS exam, aiding users in enhancing their vocabulary repertoires and schematic knowledge on a wide range of topics. It also builds up learners’ main strategies and skills they require for listening module of the exam.

#### Oxford placement test (OPT)

To measure participants’ general language proficiency, OPT (Allan, 2004) was administered to the participants of both groups. OPT is widely used to measure English proficiency of different learners with various proficiency levels. The reliability coefficient of OPT, as measured by Cronbach’s alpha, was.86 in this research. The results of an independent-samples *t*-test indicated that there was no substantially significant difference between the two groups, implying that the two classes were homogeneous with respect to their global English proficiency prior to beginning the treatment.

#### Listening performance test

International English Language Testing System (IELTS) listening section was used to guage the listening comprehension of the EFL students before (as pre-test) and after (as post-test) the treatment. In order to avoid test effect as the potential internal validity threat (Ary et al., 2018), two samples were adapted from the IELTS listening practice tests (Scovell et al., 2004) and used as pre-and post-tests. The students were required to answer 40 questions devoted to four sections of listening module within 30 min. The Cronbach’s alpha coefficients measured for the pre-and post-tests were.81 and.84, respectively.

#### Listening anxiety scale

Foreign Language Listening Anxiety Scale (FLLAS) was administered to assess listening anxiety of the EFL students before and after the treatment. FLLAS designed by Kim (2000) is a 33-item self-report measure in which the items are assessed on a 5-point Likert-type scale. This scale measures three facets of foreign language anxiety including *tension and worry* (10 items), *lack of confidence* (7 items), and *problems encountered* (16 items). The internal consistency of the FLLAZ, as calculated by Cronbach’s alpha formula, was.84 in the current research.

### Procedure

The students had enrolled in an advanced listening course whose purpose was to boost listening competencies of the EFL participants to get ready to take IELTS. In the first session of the course, the participants were informed of the purpose of this study and the pre-tests (i.e., listening test and listening anxiety scale) were administered to the participants of the two groups. Then the two intact classes were randomly assigned to an experimental group and a control group. The treatment was carried out in winter of 2022 and lasted for 13 sessions. Given the purpose of this study, the experimental group were taught based on the flipped mode of instruction in which the students were provided with previously prepared materials of the listening instruction before attending the class. The learning materials of the flipped group were video clips selected from YouTube, the recorded classes of previously held online courses, and Voice-annotated PowerPoints. The duration of video clips was about 20–30 min. The students were requested to go through the video clips/PowerPoints of each session before attending the class. In order to ensure that they have covered and understood the flipped content, the teacher provided the students of the experimental group with worksheets and exercises related to the videos of each session. During each session, the teacher ensured that the learners had watched and understood the content of the videos *via* collaborative tasks, worksheet completion, and group discussions. Each unit of the textbook had a three-part structure and each session lasted for about 75 min.

On the other hand, the control group students were taught traditionally without flipping any content of the course. These students received the same type of instruction and tasks but they had less time for the practice inside the classroom. The same instructor was assigned as the teacher of both classes. The listening comprehension exercises were done collaboratively by the control group students and they received teacher scaffolding if they needed. However, as they had not been provided with the course content in advance, they had less opportunity to get exposed to the audio texts as the flipped group did. In general, the control group had less time for the listening exercises and group discussions inside the class. Students of both groups were encouraged not to use other materials except for those given by the instructor. It is worth noting that much attempt was made to offer the two groups with identical instruction *via* using the same materials. However, the flipped group were endowed with further opportunity to get exposed to and review the learning materials before the classroom, leading to a kind of unparalleled teaching for the two groups. This pitfall, nevertheless, was partially mitigated by encouraging the students of the control group to undertake further listening practice outside the classroom.

The instructional content of both groups was mainly directed to strategies on how to do the test tasks of IELTS listening section. It is worth mentioning that the participants of both groups were also encouraged not to study any other textbook/materials during the course time. The teacher tried to create an inspiring learning context and enhance students’ motivation *via* pair and group exercises. He also helped students acquire effective learning strategies *via* doing tasks and having reflection on them. At the end of the course, the students of both groups were given the post-tests of listening achievement and listening anxiety to explore the potential effects of flipped listening instruction on the dependent variables of the study.

### Data analysis

The collected data were fed to SPSS (version 23.0) for data analysis. First, descriptive statistics (means and standard divisions) was taken into account. Then One-way between-groups analysis of covariance (ANCOVA) was conducted to analyze the quantitative data as the inferential statistics. Following Pallant (2020), ANCOVA can be utilized in case there is a pre-test/post-test design in which the pre-test scores are considered as the covariates. In the ANCOVA analyses, the independent variable was the teaching type (i.e., flipped or traditional) and the dependent variables were post-test scores of listening performance and listening anxiety at the end of treatment.

## Results

First, descriptive statistics was calculated. Then, to explore the impact of the flipped listening course on the EFL learners’ listening performance and listening anxiety, the analysis of covariance (ANCOVA) was performed to compare the impacts of the two kinds of listening instructions used in the control (non-flipped) group and the experimental (flipped) groups on the listening performance and listening anxiety.

Prior to performing each ANCOVA, preliminary investigations were done to make sure that the assumptions of normality, linearity, homogeneity of variances, homogeneity of regression slopes, and reliable measurement of the covariate were not violated.

Table 1 shows the skewedness and kurtosis statistics and their ratios to the standard errors. As the ratios fell between –1.96 and + 1.96, the data were regarded to be normally distributed.

**Table 1:** Test of normality.

	N	Skewedness (Std. Error)	Kurtosis (Std. Error)
Listening 1	44	0.456 (0.314)	−0.356 (0.658)
Listening 2	44	0.431 (0.314)	−1.102 (0.658)
Anxiety 1	44	−0.356 (0.314)	−0.174 (0.658)
Anxiety 2	44	−0.336 (0.314)	−0.496 (0.658)

Moreover, the Levene’s test for homogeneity of variance for listening performance scores showed the equality of variances as no significant difference was found between the variance of the groups (*F* = 4.65, *p* = 0.353). Additionally, it was found that the interaction between the covariate (pre-test scores of listening) and independent variable (instruction type) was insignificant (*F* = 33.54, *p* = 0.412). Likewise, homogeneity of variance (i.e., Levene’s test) was assessed for listening anxiety scores. The results demonstrated no significant difference between the variance of the groups (*F* = 3.89, *p* = 0.326). The interaction between the covariate (pre-test scores of listening anxiety) and independent variable was not also significant (*F* = 28.27, *p* = 0.357). These results confirmed that the assumptions of ANCOVA were met, leading the researchers to conduct ANCOVAs for both listening performance and listening anxiety.

Concerning the impact of the flipped listening instruction on listening performance of EFL learners, as Table 2 indicates, the listening performance mean score of the experimental group was 17.79 (SD = 4.01) on the pre-test and it increased to 23.24 (SD = 4.53) on the post-test. By the same token, the mean score of the listening performance for the control group on the pre-test was increased from 18.14 (SD = 3.30) to 19.46 (SD = 4.24) on the post-test. Nevertheless, after adjusting for the pre-test scores of listening performance, a statistically significant difference was found between the two groups on post-test scores of listening performance (*F*(1, 41) = 10.60, *p* = 0.002, partial eta squared = 0.20; see Table 3). This finding indicates that the participants of the flipped group enhanced their listening performance significantly more than the non-flipped group participants, implying that the flipped listening instruction was effective in improving the listening performance of the EFL participants.

**Table 2:** Descriptive statistics for pre-and post-tests scores.

		Pre-test		Post-test	
**Groups**	**Scales**	***M***	***SD***	***M***	***SD***
Experimental	Listening	17.79	4.01	23.24	4.53
	Anxiety	44.04	10.85	36.69	10.65
Control	Listening	18.14	3.30	19.46	4.24
	Anxiety	46.30	11.70	43.57	11.43

**Table 3:** ANCOVA results for listening performance.

Source	Type III Sum of Squares	df	Mean Square	F	Sig.	Partial Eta Squared
Covariate (pre-test)	150.679	1	150.679	9.321	0.004	0.185
Between-subjects	171.425	1	171.425	10.604	0.002	0.205
Within-subjects	662.822	41	16.166			

With regard to listening anxiety, the descriptive statistics (see Table 2) demonstrate that the non-flipped group had a listening anxiety mean score of 46.30 (SD = 11.70) in the pre-test and this mean score decreased to 43.57 (11.43) on the post-test. Similarly, the listening anxiety mean score for the experimental (flipped) group was 44.04 (SD = 10.85) on the pre-test and this value was decreased to 36.69 (SD = 10.65) on the post-test. After adjusting for the pre-test scores of listening anxiety, the results of ANCOVA (see Table 4) showed that a statistically significant difference was observed between the two groups on post-test scores of listening anxiety, [*F*(1, 41) = 176.83, *p* = 0.000, partial eta squared = 0.81]. This result revealed that flipped listening instruction was effective in reducing the listening anxiety of the EFL participants.

**Table 4:** ANCOVA results for listening anxiety.

Source	Type III Sum of Squares	df	Mean Square	F	Sig.	Partial Eta Squared
Covariate (pre-test)	5059.113	1	5059.113	3778.276	0.000	0.989
Between-subjects	236.780	1	236.780	176.833	0.000	0.812
Within-subjects	54.899	41	1.339			

## Discussion

The purpose of the present research was set to deepen our knowledge of how flipped classroom can affect L2 listening competence among EFL learners in the context of China. In order to further elucidate the utility of technology and its integration into L2 learning, the current study sought to explore the impact of the flipped instructional model on listening comprehension and listening anxiety of Chinese EFL learners. The outcomes revealed two main findings. First, it was found that the flipped classroom developed the listening comprehension of the participants. This finding is on a par with a number of previous studies (Amiryousefi, 2017; Vaezi et al., 2019; Etemadfar et al., 2020; Rajabi et al., 2021) which underscored the effectiveness of the flipped classroom model in improving L2 learners’ listening comprehension. For instance, the findings from Etemadfar et al. (2020) study revealed that the EFL learners in the flipped classroom outperformed their classmates in the control group in terms of their listening comprehension. Similarly, Rajabi et al. (2021) indicated that the flipped classroom significantly improved the listening performance of the EFL participants. Likewise, Amiryousefi (2017) demonstrated that flipped learning helped EFL students to improve their L2 listening and engagement with materials outside of class. Also, this finding somewhat resonates with the result of Shahnama et al. (2021) study, suggesting that the flipped model promotes L2/EFL learners’ achievement.

In light of justifying this result, it can be argued that the flexible and autonomous nature of the flipped classroom enables learners to learn on their own pace at anyplace or anytime they prefer. Put differently, learners are able to listen to the given materials (e.g., podcast, vodcast, or instructional videos) as many times as they desire until they understand the content. Unlike the traditional teacher-centered instruction where the allocated time of the class is not enough for re-listening to the audio files, students of the flipped group can pause, rewind, and replay the materials to reinforce their listening comprehension. Students can learn different subjects in depth and become self-regulated as long as the content is shared with them prior to coming to the class. In spite of the pivotal role that listening skill plays in learning the L2 (Vandergrift, 2007), teachers sometimes underestimate the listening activities in the course books and ignore the output due to the lack of time in traditional classrooms. The reverse nature of the flipped classroom allows teachers to free up the class time for authentic listening tasks as well as output-based activities and devote their energy to giving further feedback and assessment. Consequently, learners have further opportunities for benefiting from the authentic materials, individualized feedback from their instructor, and peer support in the flipped classroom (Alten et al., 2019). Moreover, high exposure to authentic listening materials especially outside the class where learners have enough time and less anxiety to view and review the contents may develop learners’ listening comprehension. This is to say, the rich input through the instructional materials (podcasts and videos), accompanied by classroom interactions and proper feedback improves learners’ listening comprehension. Rajabi et al. (2021) hold the view that authentic materials if properly selected for different language proficiency levels can have useful impacts on L2 learners’ listening proficiency. Another possible explanation may be that since learners in the flipped model have greater chances to contemplate on and decode the meaning of different sentences in the audio files with full consideration at home, they can minimize any mistake and misunderstanding. Therefore, teachers can make use of the extra time inside the class to address misconceptions and provide students with more personalized guidance on their listening comprehension. In addition to teachers’ help and guidance, learners can also receive support from their classmates (Alten et al., 2019). More specifically, the flipped classroom is effective for students in case of being absent since they can access the instruction beyond the limitations of classroom wall.

Second, the listening anxiety of the EFL learners in the experimental group was significantly reduced after experiencing flipped instruction. This finding is partially in congruence with Abdolrezapour (2019) study which revealed that computer-mediated active learning intervention reduced English learners’ anxiety in listening tasks and enhanced their motivation to participate in classroom activities. One interpretation for the obtained result might be that in the flipped classroom students have greater opportunity to learn and perform in active/collaborative learning (Lai and Hwang, 2016) and get engaged in higher-order thinking tasks, thus they experience less anxiety. Team- or peer-assisted learning (Topping and Ehly, 1998) and interactive activities not only help students to improve their listening comprehension, but also decrease their anxiety level through sharing diverse perspectives and solving problems. This interpretation goes along with some researchers who believed that collaborative strategies that engage learners in peer-to-peer interactive tasks support L2 learning (e.g., Vandergrift, 1997) and increase learners’ comprehensible input which lead them to have greater understanding of aural texts (Szostek, 1994). When learners discuss and interact with each other, they support, guide, and correct each other when needed and, thereby helping each other to progress by sharing their knowledge, cognitive, and linguistic resources (Rowell, 2002).

Based on what has been mentioned so far, it can be argued that collaborative listening activities in the flipped classroom may develop reciprocity and cooperation among learners which result in reducing their listening anxiety. In such an environment where the students have the same learning goals and support each other in this regard, anxious learners feel less stress. It is worth noting that in contrast to conventional classrooms in which anxious students feel embarrassed to speak in front of their peers, in flipped classrooms they receive social support and encouragement from the teammates to share opinions regarding listening. As Davies et al. (2013) noted, in the flipped classroom learners are transformed from mere passive listeners into active students. Another possible explanation is concerned with the students’ preparedness before class (Basal, 2015) and enjoyment of the activities in class. The individualized nature of flipped model allows students to pre-study the materials at home without any distraction and get ready to answer the instructor’s questions inside the class. Students would take part effectively in classroom discussions because they have gained a basic background about the lesson prior to class (Kukulska-Hulme and Viberg, 2018).

According to Bergmann and Sams (2012), students are willing to apply their knowledge in details *via* various problem-solving tasks, pair/group works, and discussion-based activities. In such a case, students feel less anxiety when they encounter the questions because they have relevant prior knowledge related to the contents. Through technology, students can search numerous learning resources online, watch videos on YouTube, and do listening quizzes online before class. As Sahin et al. (2015) stated, learners had positive perceptions toward flipped learning because watching videos was easier than reading the coursebook for them. Their argument may explain why learners in flipped classrooms might be better prepared before participating in class than their counterparts in conventional instruction settings. However, this study is inconsistent with the findings of Rajabi et al. (2021) which showed that the flipped classroom has no effect on EFL learners’ classroom anxiety.

## Conclusion

In this study, we examined the effect of the flipped classroom approach on L2 listening comprehension and L2 listening anxiety of Chinese EFL students. Overall, the findings revealed that the flipped classroom had a key role in developing the participants’ L2 listening comprehension. Moreover, learners in the experimental group experienced less anxiety compared to their peers in the conventional group. Despite the fact that flipped learning has been in the spotlight among a growing number of L2 instructors, the salient role of listening competence has been ignored in ELT context. It is hoped that the results obtained from this study could yield beneficial insights into the field of EFL by employing a more collaborative genre of digital learning (i.e., flipped classroom) in which listening comprehension skill is sufficiently practiced. As Shahnama et al. (2021) posited, although the flipped learning is in its infancy in the domain of EFL, it has the potential to improve learners’ achievements in case of being designed and employed properly.

There are some implications derived from the present study. CALL programs with the goal of practicing successful flipped learning must create a friendly and welcoming social context for online EFL learning. In this respect, teachers can furnish an effective online community using various social networking sites (e.g., Telegram, Whatsapp, and so forth) in which the EFL learners can maximize their interaction and share their knowledge with regard to L2 listening. The learners can view the instructional videos deeply at a time that suits them best to diagnose their listening issues during the class activities. Thus, teachers can devote the class time to authentic output activities in order to enhance the students’ listening comprehension and give appropriate and rapid feedback. As Wang (2020) mentioned, interacting and cooperating in the online space enables the students to learn from each other and also to reflect on their own learning.

It is recommended that flipped classroom, as a relatively new instructional approach in the educational system, be applied to develop learners’ language skills and L2 listening comprehension in particular. To meet this purpose, teachers have active roles in encouraging and facilitating collaborative listening activities, peer-peer interactions, and in-class assessment. EFL instructors can give their students the necessary advice and guide them how to cope with unfamiliar vocabulary or grammatical patterns in the listening files they hear. With enough guidance and practice in a comfortable and stress-free classroom through interactive and collaborative activities, students could improve their listening comprehension skills and reduce their anxiety level too. Elkhafaifi (2005) suggested that creating a less stressful and comfortable classroom environment may enable students to improve their listening comprehension proficiency. Appropriate instruction in listening comprehension also decreases listening anxiety and provides students with a sense of autonomy. Even though teaching listening is believed to be difficult and time consuming in traditional lecture-based classrooms, the flipped teaching approach makes it easier and more pleasant for both teachers and students. Drawing on the tenets of positive psychology (Wang et al., 2021) can also help teachers to create a positive teacher-student interpersonal relations (Xie and Derakhshan, 2021). Given that most of the EFL teachers pursue traditional pedagogical methods in their lecture-based classes, they are unfamiliar with the utility of Web 2.0 technology in general. Therefore, EFL policymakers and teacher educators should hold teacher trainings workshops to inform the instructors of the procedures required to conduct a successful flipped listening classroom to contribute to EFL learners’ listening comprehension. They might also create a convenient education environment for EFL instructors and learners so that they may have a chance to practice their listening skills in a flipped classroom.

The current study suffers from some limitations that need to be taken into account for future research. First, this research recruited a small number of EFL learners; therefore, generalizability of the results may not be verified. Second, since this study was carried out in the EFL context of China, the findings might not be transferable to other EFL populations. As a result, a cross-cultural survey may be appropriate to provide a more vivid picture of the flipped model in different settings where English is considered as a FL. Also, next researchers are recommended to employ qualitative research methods too. Employing qualitative research designs will allow the researchers to get more insight into what practical and logistical challenges the instructors and the students might have experienced when the flipped method was implemented. Similarly, the EFL learners’ perceptions regarding their experience of flipped instruction can be qualitatively explored in order to investigate how much qualitative results confirm the quantitative findings. In addition, in the current study, we just investigated the students’ listening development and anxiety through pre-and post-tests. Having replicated the similar further study, future researchers may conduct delayed post-tests to recognize the long-term impacts of the flipped classroom on listening comprehension among EFL students. The EFL teachers’ attitudes and outlook toward the flipped classroom integration for listening skill would be a good research topic for future studies. Additionally, attempts need to be made also to find the influential and useful strategies to improve students’ listening comprehension skill in flipped classroom evaluation and ways to encourage students to view the materials out of class and participate in-class activities. Ultimately, as the individual differences and the effect of cultural, educational background, and other contextual factors can potentially affect the internal validity of such studies, future researchers might design studies to examine the role of these extraneous variables by considering them as moderator variables or to carry out cross-cultural studies.

## Data availability statement

The raw data supporting the conclusions of this article will be made available by the authors, without undue reservation.

## Ethics statement

The studies involving human participants were reviewed and approved by Lingnan Normal University Research Ethics Office. The patients/participants provided their written informed consent to participate in this study.

## Author contributions

All authors listed have made a substantial, direct, and intellectual contribution to the work and approved it for publication.

## Conflict of interest

The authors declare that the research was conducted in the absence of any commercial or financial relationships that could be construed as a potential conflict of interest.

## Publisher’s note

All claims expressed in this article are solely those of the authors and do not necessarily represent those of their affiliated organizations, or those of the publisher, the editors and the reviewers. Any product that may be evaluated in this article, or claim that may be made by its manufacturer, is not guaranteed or endorsed by the publisher.
